# Morphology and Histochemistry of the Aesthetasc-Associated Epidermal Glands in Terrestrial Hermit Crabs of the Genus *Coenobita* (Decapoda: Paguroidea)

**DOI:** 10.1371/journal.pone.0096430

**Published:** 2014-05-07

**Authors:** Oksana Tuchina, Katrin C. Groh, Giovanni Talarico, Carsten H. G. Müller, Natalie Wielsch, Yvonne Hupfer, Aleš Svatoš, Ewald Grosse-Wilde, Bill S. Hansson

**Affiliations:** 1 Department of Evolutionary Neuroethology, Max Planck Institute for Chemical Ecology, Jena, Germany; 2 Department of Forensic Toxicology, Institute of Legal Medicine, University of Greifswald, Greifswald, Germany; 3 Department of Cytology and Evolutionary Biology, Ernst-Moritz-Arndt-University, Zoological Institute and Museum, Greifswald, Germany; 4 Department of Neuroscience, University of Arizona, Tucson, United States of America; 5 Research Group of Mass Spectrometry and Proteomics, Max Planck Institute for Chemical Ecology, Jena, Germany; Hospital Nacional de Parapléjicos – SESCAM, Spain

## Abstract

Crustaceans have successfully adapted to a variety of environments including fresh- and saltwater as well as land. Transition from an aquatic to a terrestrial lifestyle required adaptations of the sensory equipment of an animal, particularly in olfaction, where the stimulus itself changes from hydrophilic to mainly hydrophobic, air-borne molecules. Hermit crabs *Coenobita* spp. (Anomura, Coenobitidae) have adapted to a fully terrestrial lifestyle as adults and have been shown to rely on olfaction in order to detect distant food items. We observed that the specialized olfactory sensilla in *Coenobita*, named aesthetascs, are immersed in a layer of mucous-like substance. We hypothesized that the mucous is produced by antennal glands and affects functioning of the aesthetascs.

Using various microscopic and histochemical techniques we proved that the mucous is produced by aesthetasc-associated epidermal glands, which we consider to be modified rosette-type aesthetasc tegumental glands known from aquatic decapods. These epidermal glands in *Coenobita* are multicellular exocrine organs of the recto-canal type with tubulo-acinar arrangement of the secretory cells. Two distinct populations of secretory cells were clearly distinguishable with light and electron microscopy. At least part of the secretory cells contains specific enzymes, CUB-serine proteases, which are likely to be secreted on the surface of the aesthetasc pad and take part in antimicrobial defense. Proteomic analysis of the glandular tissue corroborates the idea that the secretions of the aesthetasc-associated epidermal glands are involved in immune responses.

We propose that the mucous covering the aesthetascs in *Coenobita* takes part in antimicrobial defense and at the same time provides the moisture essential for odor perception in terrestrial hermit crabs. We conclude that the morphological modifications of the aesthetasc-associated epidermal glands as well as the functional characteristics of their secretions are important adaptations to a terrestrial lifestyle.

## Introduction

Among hermit crabs (Decapoda, Anomura), Coenobitidae represents a perfect model to study the impact of terrestrialization on the structure and function of the olfactory system in invertebrates. From their earliest appearance in coastal habitats, which was 20 Mya according to the fossil record [1], [2], coenobitids have adapted to a fully terrestrial lifestyle as adults [3]. The ability to permanently live ashore and locate distant food items or shell sources relies heavily on olfaction [4]–[6]. An essential prerequisite of good olfactory sense is the development of proper olfactory centers, which in coenobitids dominate the brain [7]. Long-distance detection of odors takes place on the external distal ramus of the antennules, called flagellum, which bears several rows of specialized olfactory sensilla, the aesthetascs [8]. In *Coenobita clypeatus*, each sensillum is peg-shaped and houses multiple ramified dendrites of approximately 300 olfactory sensory neurons [8], [9]. The sensory cell somata are arranged in spindle-like complexes [8]. The thin cuticle of the exposed surface of the aesthetascs does not display any obvious pores, as is well-known for some types of olfactory sensilla of various myriapods and insects [10]–[12]. Thus, the question how airborne odor molecules penetrate the aesthetasc cuticle in crustaceans is not answered yet. However, numerous openings in the cuticle closely associated with the aesthetascs have been found in marine decapods [13]. These pores were identified as openings of epidermal exocrine glands, also referred to as aesthetasc tegumental glands in *Panulirus argus* (Latreille, 1804), here abbreviated as ATGs [13]. The secretory cells in the ATGs of *P. argus* were shown to contain a specific type of proteases – CUB-serine proteases (Csp) [13]. Serine proteases are a diverse family of trypsin- and chymotrypsin-like enzymes, which contain two amino acid residues, histidine and aspartate, to stabilize the third active center, serine; together they form a catalytic triad acting on a substrate [14]. CUB is an extracellular protein domain, named after three proteins from which it was first identified: complement subcomponents Clr/Cls, embryonic sea urchin protein, Uegf, or fibropellin, and bone morphogenetic protein1, Bmpl [15]. CUB possesses four conserved cysteine residues, which form two disulfide bridges [15], [16].

We wondered whether hermit crabs of the genus *Coenobita* also possess aesthetasc-associated epidermal glands and corresponding porous structures in their antennules.

Arthropod epidermal exocrine glands are well-known in crustaceans and may consist of a single secretory cell (class-I-glands according to Quennedey, [17]) or several specialized cell types, such as secretory cells and canal cells which guide the secretion outside via a cuticularized duct (class-III-glands [17]). Class-III-glands may remain solitary or may form glandular organs of higher complexity [12], [18]. Most recently, Müller et al. [12], [18], [19] distinguished two subclasses of class-III-glands, flexo-canal and recto-canal epidermal glands; both types share an intermediary cell linking the secretory cell(s) and canal cell(s). Flexo-canal glands are characterized by meandering (flexuous) part of the conducting canal [19], while the recto-canal glands have straight-running conducting canals which locally widen to form huge reservoirs. Recto-canal glands are widely distributed among crustacean taxa and show great structural and functional diversity (reviewed in [18], [20]). Glandular units may contain two, three or numerous cells. In the latter case, secretory, intermediary, and canal cells are often clustered in rosette-like formation; syncytial and lobed glandular complexes have also been reported reaching deeply below the epidermis of the head, mouth parts, pleopods, eyestalks, midgut and antennae in many different crustacean species (see [20], [21] for details). The ubiquity of epidermal glands suggests that they play a pivotal role in the interaction of an organism with the environment. However, the exact function of investigated epidermal glands remains unknown in most cases.

Here, we set out to analyze the antennular glands of *Coenobita* species in a broad methodological approach ranging from an ultrastructural investigation comprising SEM and TEM techniques to standard methylene blue histology, histochemistry with nuclear marker sytox green, application of F-actin marker Phalloidin, cLSM imaging of fluorescent dyes around the conducting canals of the glands, applying backfilling techniques, immunohistochemistry with CUB-serine protease antibodies, and proteomic analysis of the glandular tissue. Besides providing a thorough description of the anatomy of the antennular glands, we also intend to gain insights into possible function of these glands in *Coenobita*.

## Materials and Methods

### Animals

This study combines observations on the first antennae (antennules) of *Coenobita clypeatus* (Herbst, 1791), *C. scaevola* (Forskål, 1775) and *C. compressus* (H. Milne-Edwards, 1836). *C. clypeatus* was obtained from the “Zoologischer Groβhandel Peter Hoch” (August Jeanmaire Str. 12, 79183 Waldkirch, Germany; http://www.hoch-rep.com/). *C. compressus* was collected nearby Playa Naranjo in Santa Rosa Nacional Parque, Costa Rica, in May 2008. Permits were given by SINAC and MINAE, respectively (“Resolucion de investigacion cientifica”, document: ACG-Pl-021-2008). *C. scaevola* was collected on the beach of Dahab (Sinai Peninsula, Egypt). Permits were provided by EEAA. Prior to all dissections, animals were anesthetized on ice for 10–30 min and removed from their shells.

### Scanning electron microscopy (SEM)

SEM was performed on the large flagella of two specimens of *C. clypeatus*. After rinsing animals with tap water, the antennules were removed; their large flagella were cut off and were immediately placed in 50% ethanol. All samples were dehydrated in a graded series of ethanol (60, 70, 80% two times 10 min each; 90%, 96% for 10 min each; absolute EtOH overnight), critical-point-dried using a BAL-TEC CPD 030, mounted on aluminum stubs with adhesive film, and sputter-coated with gold on a BAL-TEC SCD005 prior to examination with a LEO 1450 VP scanning electron microscope, operated at 10 kV (Carl Zeiss AG, Oberkochen, Germany).

### Transmission electron microscopy (TEM)

TEM was performed on the large flagella of *C. clypeatus*, *C. scaevola* and *C. compressus*. Two antennules of *C. clypeatus* were cut off and their large flagella were dissected to smaller subunits in cold fixative (2.5% glutaraldehyde either in phosphate or cacodylate buffer) and prefixed for 12 h at 4°C in the same solution. Samples were rinsed three times for 10 min with chilled buffer and postfixed in solutions containing either 2% osmium tetroxide (OsO_4_) in phosphate buffer or 1% OsO_4_ in cacodylate buffer for 2 h at 4°C. After rinsing three times for 10 min with chilled buffer, the samples were dehydrated in graded series of ethanol (see above). Dehydrated samples were embedded in epoxy resin (Durcupan) and polymerized for 20 h at 65°C. 30 antennules of *C. compressus* and 12 of *C. scaevola* were fixed in a cold solution modified after Karnovsky [22] containing 2.5% glutaraldehyde, 2.5% paraformaldehyde, 1.5% NaOH, and 1.2 g D-glucose dissolved in 2.25% sodium phosphate buffer (adjusted to pH 7.4) for 6 h (*C. compressus*) and 7d (*C. scaevola*), afterwards antennules were washed several times in the same buffer solution, dehydrated in a graded series of ethanol, and finally embedded in epoxy resin (Araldite). Ultrathin sections at thickness of 50 to 70 nm were cut with a Diatome diamond knife (Ultra 35°) on a Reichert Ultracut microtome. Sections were collected on Pioloform-coated single slot copper grids and examined without additional staining with a Zeiss CEM 902A (with a TVIPS FastScan digital camera) transmission electron microscope, operating at 10 kV (Carl Zeiss AG, Oberkochen, Germany).

### Light microscopy (LM)

For orientation, semithin sections (150 to 400 nm in thickness) were transferred to glass-slides and stained with methylene blue-borax/Azure II according to Richardson et al., [23]. Sections were observed either on a Zeiss AxioImager Z.1 with an AxioCam HRc (Carl Zeiss AG, Jena, Germany) or on a Zeiss Axioscope 50 (Carl Zeiss AG, Jena, Germany) with a PixeLINK PL-B623CF-KIT 3.0 MP FireWire Camera (PixeLINK, Ottawa, Canada).

### Confocal laser-scanning microscopy (cLSM)

In order to fully visualize the general distribution of the glandular ducts a crystal of micro-ruby (dextran tetramethylrhodamine-biotin, MW 3000, lysine fixable, Invitrogen) was inserted into the third annulus of the antennular flagellum using a glass capillary. After at least 3 h of exposition to the dye, antennules were cut and immediately fixed with 4% paraformaldehyde (PFA) in phosphate buffered saline (PBS, pH 7.3), for no less than 2 h at room temperature on a shaker or overnight at 4°C. After fixation, tissues were washed in several changes of PBS for 2 h in total, the cuticle was entirely or partially removed, and then tissues were dehydrated in a graded series of ethanol (50%, 70%, 80%, 2×99%, 15 min each), finally immersed in methyl salicylate and observed under the fluorescence stereomicroscope Leica MZ16FA (Leica Microsystems GmbH, Wetzlar, Germany) and the laser-scanning microscope Zeiss LSM 510 Meta (Carl Zeiss GmbH, Jena, Germany). Pictures were obtained using LSM image browser.

### Histochemistry

We used phalloidin for selective staining of actin in the canal cells of the glands of 15 specimens of *C. clypeatus*, either alone or combined with nuclear stain (i.e. double-staining). Antennules were first fixed in 4% PFA in PBS for 2 h at room temperature on a shaker, then washed in several changes of PBS with 0.5% Triton X (PBST) for no less than 2 h and finally incubated in phalloidin Alexa 546 (1∶50; Molecular Probes) overnight at 4°C on a shaker. After incubation, tissues were washed again in PBST and in case of double-staining incubated with sytox green (1∶1000; Invitrogen). During incubation with sytox, tissues were checked time to time under the fluorescence stereomicroscope Leica MZ16FA (Leica Microsystems, Wetzlar, Germany) until the degree of staining of cell bodies was considered sufficient (usually approx. 20 min). Sometimes, a partial removing of the antennular cuticle was needed for better penetration of the dyes. Then, tissues were washed in PBS for at least 30 min and further processed in a graded series of ethanol and methyl salicylate as described above.

### Csp immunoreactivity

The polyclonal Csp antibodies (5^th^ bleed serum [99-5]) generated against the CUB-domain of *Panulirus argus* serine protease) were kindly provided by Prof. Dr. Manfred Schmidt and Prof. Dr. Charles D. Derby (see [13], [16]). To test for Csp immunoreactivity, antennules of 5 specimens of *C. clypeatus* were first fixed in fresh 4% PFA as described above, and washed in several changes of PBS. The cuticle was removed completely and antennular tissues (whole mounts) were processed according to standard protocol for immunohistochemical labeling (see [7]). Tissues were incubated overnight in 4% NGS (Normal goat serum, Invitrogen) on PBS-Tx (0.5% TritonX, Sigma-Aldrich), washed several times in changes of 0.5% NGS on PBS-Tx and then incubated with primary antibody (5th bleed serum [99-5] rabbit, 1∶400) for 3d at 4°C. After antibody incubation the tissues were rinsed several times in changes of 0.5% NGS on PBS-Tx and then incubated with the secondary antibody (donkey anti-rabbit Cy3, Jackson ImmunoResearch Lab, 1∶1000) overnight. Subsequently, the tissues were washed in PBS for at least 2 h and incubated with nuclear marker sytox green (Invitrogen, 1∶500) for 30 min, then washed in PBS for at least 1 h, dehydrated in a graded series of ethanol (70, 80, 90, 2×100%, 15 min each), cleared in methyl salicylate and studied under the confocal laser-scanning microscope. Simultaneously, we performed a control experiment by incubating the tissues for 3d in 0.5% NGS PBS-Tx, but omitted incubation with primary antibodies. Several antennules prepared for Csp-immunoreactivity experiments were left with their cuticle attached. These samples were cut in pieces of 2-3 annuli width and incubated with 10% EDTA for 1 week, with daily changes of the incubation solution (according to [13]), to test whether the incubation with EDTA affects staining. However, no differences have been observed between the samples incubated with EDTA and those which cuticle was removed mechanically by forceps.

### Bioinformatics – BLAST searches and alignments

BLAST searches were carried out after dynamic translation (BLASTX) with a default E-value cutoff of 1.0E-6. Alignments were compiled using the MUSCLE alignment tool [24].

### Sample preparation for proteomic analysis

The antennules of 30 anesthetized specimens of *C. clypeatus* were dissected in Lysisbuffer (7 M Urea, 2 M Thiourea, 2% 3-[(3-cholamidopropyl)dimethylammonio]-1-propanesulfonate [CHAPS], 20 mM Tris(hydroxymethyl)-aminomethan [Tris]) containing 1 mM protease inhibitor (4-(2-Aminoethyl)benzensulfonylfluorid [AEBSF]). Two kinds of samples were taken: 1) the glandular tissue, 2) the antennular tissue containing olfactory sensory neurons and no glandular complexes (control sample). Both samples were homogenized in 1 ml Lysisbuffer for 3×20 sec with 10 sec intervals with Precellys Tubes Ceramic Beads (Precellys, 1.4 mm). The obtained lysate was transferred to polycarbonate-centrifuge vials (BECKMAN), and ultracentrifuged for 45 min at 100,000 g at 4°C. The supernatant was collected, concentrated using speed vac and 30 ul and used for SDS-PAGE.

### In-gel tryptic digestion and peptide extraction

Purified proteins were separated by 1-D SDS-PAGE and visualized by staining with Coomassie Blue (R250). Protein bands were excised from the gel, cut into small slices, washed several times with 25 mM ammonium bicarbonate and destained with 50% ACN/25 mM ammonium bicarbonate. Disulfide bonds were reduced with 10 mM DTT at 50°C for 1 h and alkylated with 55 mM IAA at room temperature in the dark for 45 min. Following tryptic digestion in 0.5 µM solution (in 25 mM ammonium bicarbonate) of porcine trypsin (Promega) overnight at 37°C, the peptides were extracted from the gel pieces using 75% ACN/5% formic acid (FA), and dried down in a vacuum centrifuge [25]. For mass spectrometric analysis samples were reconstructed in 10 µL aqueous 0.1% FA.

### Nano-UPLC-MS/MS analysis

The peptide mixtures (1 to 8 µL) were initially concentrated and desalted on a Symmetry C18 trap-column (20×0.18 mm, 5 µm particle size, Waters) using 0.1% FA as mobile phase at a flow rate of 15 µL/min. Then, peptides were separated on a nanoAcquity C18 analytical column (200 mm×75 µm ID, C18 BEH 130 material, 1.7 µm particle size, Waters) using an increasing acetonitrile gradient in 0.1% FA at a flow rate of 350 nl/min. The applied LC-gradient was: 1–30% B over 13 min, 30–50% B over 5 min, 50–95% B over 5 min, isocratic at 95% B for 4 min, and a return to 1% B over 1 min (phases A and B composed of 0.1%FA and 100% ACN in 0.1% FA, respectively); the analytical column was re-equilibrated for 9 min prior to the next injection.

The eluted peptides were on-line transferred via a NanoLockSpray ion source into a Synapt HDMS tandem mass spectrometer (Waters). The source temperature was set to 80°C, cone gas flow 30 L/h, and the nanoelectrospray voltage was 3.2 kV. For all measurements, the mass spectrometer was operated in V-mode with a resolution power of at least 10,000 FWHM. All analyses were performed in positive ESI mode. The lockmass calibrant standard, human Glu-Fibrinopeptide B (650 fmol/mL in 0.1% FA/ACN (1∶1 v/v)), was infused into the NanoLockSpray electrospray source at a flow rate of 500 nL/min through the reference sprayer every 30 sec to compensate for mass shifts in MS and MS/MS fragmentation mode.

LC-MS data were collected using MassLynx v4.1 software under data-dependent (DDA) and data-independent (DIA)/LC-MS^E^ acquisition. For DDA, the acquisition cycle consisted of a survey scan covering the range of m/z 400–1700 Da followed by MS/MS fragmentation of the four most intense precursor ions collected at 1 sec intervals in the range of 50–1700 m/z. Dynamic exclusion was applied to minimize multiple fragmentations for the same precursor ions. For LC-MS^E^ analyses, full-scan LC-MS data were collected using alternating mode of acquisition: low energy (MS) and elevated energy (MS^E^) mode at 1.5 sec intervals with a 0.2 sec inter-scan delay in the range m/z of 300–1900 and 50–1700, respectively. The collision energy of low energy MS mode and high-energy mode (MS^E^) were set to 4 eV and 15–40 eV energy ramping, respectively.

### Data processing and protein identification

ProteinLynx Global Server (PLGS) version 2.5.2 (Waters) was used for processing of raw files and for database searching. DDA raw files were initially baseline subtracted, smoothed, deisotoped, lock-mass corrected, and pkl-files were generated. Processed MS/MS spectra (pkl-files) were searched against the NCBI-nr database (updated on December 5, 2012, containing 21,786,050 sequence entries) combined with the *Coenobita* protein subdatabase (containing 300,210 entries, constructed from an in-house created EST database by its translation from all six reading frames [26] using MASCOT v2.4 software installed on a local server. Trypsin was set as the primary digest reagent, and one missed trypsin cleavage site was allowed. Mass tolerances for precursor and fragment ions were 15 ppm and 0.03 Da, respectively. A fixed modification of carbamidomethyl-Cys was specified, and oxidation-Met was set as a variable modification. Proteins matched by at least three peptides with ion scores above 30 or by one peptide with protein score of higher than 55 were considered as correct assignments.

In parallel, MS/MS spectra were searched using PLGS software as a search engine against a subdatabase containing common background proteins (human keratins and trypsin) and the unassigned spectra were sequenced *de novo*. *De Novo* sequencing was performed using following parameters: mass deviation, 0.002 Da, and ladder score>40. Originated peptide sequence proposals were subjected to sequence-similarity searching using the MS BLAST program installed on an in-house server. MS BLAST searches were performed against the complete NCBI-nr database (updated on December 5, 2012) as well as three subdatabases: *arthropoda*, *bacteria* (both downloaded from NCBI-nr, containing 853,629 and 13,530,307 sequence entries, respectively) and *Coenobita* (constructed as described above) using adopted settings parameters [27]. Statistical significance of the hits was evaluated according to the MS BLAST scoring scheme [27].

The continuum LC-MS^E^ data were lock-mass corrected, smoothed, background subtracted, centered, deisotoped, and charge state reduced using PLGS software. Product ion spectra were generated using following thresholds for low/high energy scan ions and peptide intensity: 150, 30, and 750 counts, respectively. Time-based alignment of precursor and fragment ions was done using Ion Accounting Algorithm as described [28]. Processed data were searched against the constructed Coenobita database (as described above) combined with the Swissprot database (downloaded on July 27, 2011 from http://www.uniprot.org/). Database searching was restricted to tryptic peptides with a fixed carbamidomethyl modification for Cys residues, along with variable oxidation of Met. Further, default searching parameter specifying mass measurement accuracy were used, minimum number of product ion matches per peptide (5), minimum number of product ion matches per protein (7), minimum number of peptide matches (2), and maximum number of missed tryptic cleavage sites (1). Maximum false positive rate was set to 2% and all peptides matched under the 2% FDR were considered as correct assignments.

All identified proteins of each dataset were combined in one list, removing duplicates. The list of proteins identified in the control section was subtracted from the list of proteins from the gland section; resulting in a list of proteins only present in the sample containing glandular complexes. Entries where contigs but no homologue was assigned based on peptides were searched against non-redundant databases of the National Center of Biotechnology Information (NCBI) after dynamic translation (BLASTX). BLAST results were added where applicable (E-Value<10^−6^) (full list in Additional file 3). Entries were assigned to Gene Ontology terms in the category “Molecular Function” by manual search (http://www.geneontology.org/).

## Results

### Antennules, large flagella and aesthetasc pads

**Figure 1:**
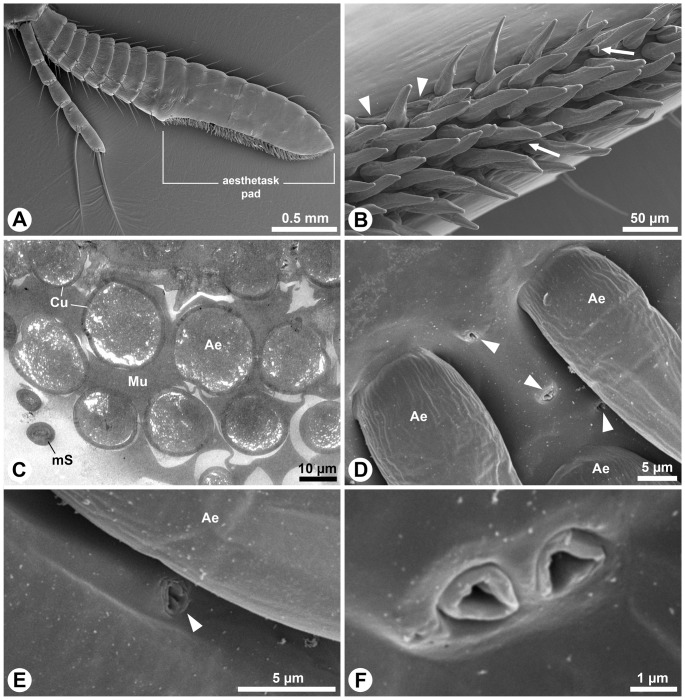
External (A–B, D–F) and internal (C) morphology of flagella, aesthetascs and antennal glands of *Coenobita*. **A**–**B, D**–**F**: SEM; **C**: TEM. **A**: Lateral view on the pair of flagella of an antennule of *C. clypeatus*. The larger dorsal flagellum carries the aesthetasc pad. **B**: Ventral view of the median part of the aesthetasc pad showing six rows of slightly bent, peg-shaped aesthetascs. Note minute setae at the lateral margin of the aesthetasc pad (arrowheads) and between the aesthetascs (arrows). **C**: Transverse section of the aesthetasc pad of *C. scaevola* not treated with fixative solutions. Mucous is present in the interspace of the aesthetascs. **D**: Detail of proximal margin of the aesthetasc pad in *C. clypeatus*. Note scattered gland pores (arrowheads) between adjacent aesthetascs. **E**: Solitary gland pore (arrowhead) from the lateral margin of the aesthetasc pad. **F**: Paired gland pore, note collar-like fold surrounding the opening. Ae, aesthetasc; Cu, cuticle; mS, minute seta; Mu, mucous.

The antennules of decapods terminate in two morphologically distinct flagella. The larger one represents the lateral and the smaller one represents the medial flagellum of aquatic species. Terrestrial hermit crabs (Coenobitidae) predominantly hold and move their antennules in a way that the large flagellum is located dorsally, while the small flagellum is oriented ventrally. The large flagellum carries a distal pad equipped with peg-shaped olfactory sensilla, the aesthetascs, sitting on its ventral surface (Figure 1A). The aesthetascs are arranged in 5–7 rows (Figure 1B). The total number of aesthetascs per flagellum depends on the age and the size of an animal. The fine tips of the slightly bent aesthetascs predominantly point towards the tip of the flagellum (Figure 1B). Slender, minute setae occur at the lateral margins of the aesthetasc pad (Figure 1B–C, arrowheads) and are also sparsely distributed among the rows of aesthetascs (Figure 1B, arrows).

The aesthetascs are immersed in a layer of clear liquid, further referred to as “mucous-like substance”; the layer is clearly visible in observations using high power stereo microscopy. Ethanol treatment for SEM preparation fully removes this substance. Then, the surfaces of the observed SEM samples are apparently clean without displaying any conspicuous residues (compare Figure 1A–B, D–F). Applying the fixation protocol for TEM apparently supported better preservation of the mucous-like substance or components of it, because some of the observed samples reveal the presence of an osmiophilic substance surrounding and covering the aesthetascs (Figure 1C).

### Organization and ultrastructure of aesthetasc-associated epidermal glands

#### Gland pores, canal cells and distal part of conducting canal

**Figure 2:**
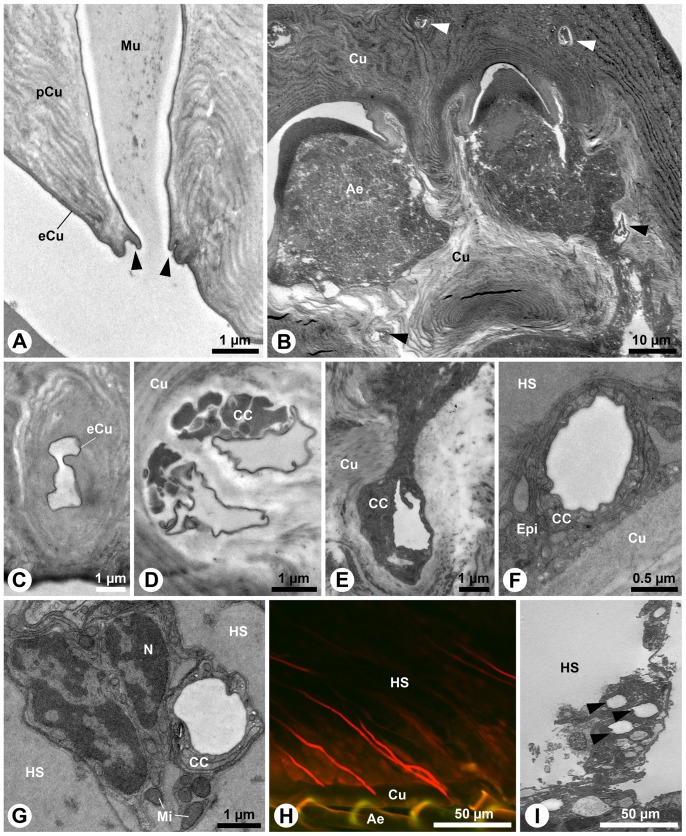
Aesthetasc-associated epidermal glands in : TEM; **H**: cLSM. **A**: Longitudinal section of a glandular pore of *C. compressus*. Note the collar-like fold (arrowheads). **B**: Horizontal section of aesthetasc pad of *C. clypeatus* showing two aesthetasc sockets and several cross-cut gland part of the conducting canal (arrowheads). **C**–**G**: Series of transverse sections showing the distal part of conducting canal on different section levels (from distal to proximal). **C**: One single duct passing through the cuticle of *C. clypeatus* closely below the glandular pore. **D**: Paired glandular ducts accompanied by cytoplasmic protrusions of the canal cells, on proximal level of the flagellar cuticle. *C. clypeatus*. **E**: Gland duct below the flagellar cuticle in *C. clypeatus*. **F**: Gland duct of *C. compressus* passing through the thin epidermis below the flagellar cuticle. The duct lumen is lined by thin epicuticle (cuticular intima). **G**: Duct passing through the hemolymphatic space in *C. compressus*. **H**: Optical sagittal section of the large flagellum of *C. clypeatus* showing several gland ducts (red; backfilling with tetramethylrhodamine dextran) in parallel orientation. **I**: Gland ducts of *C. clypeatus* (arrowheads). Ae, aesthetasc; CC, canal cell; Cu, cuticle; eCu, epicuticle; Epi, epidermis; HS, hemolymphatic space; Mi, mitochondrion; Mu, mucous; N, nucleus; pCu, procuticle.

High-power scanning electron micrographs demonstrate the presence of numerous pores in the cuticle between the aesthetascs (Figure 1D, arrowheads) and on the lateral margins of the pad (Figure 1E). Albeit displaying different shapes, each gland pore possesses a collar-like fold surrounding the approx. 1 µm wide opening (Figure 1D–F). The pores occur predominantly alone (Figure 1D, E), but also paired arrangements are present (Figure 1F). The collar-like fold is formed by the cuticle protruding into the pore lumen (Figure 2A).

The thin, strongly electron-dense epicuticle extends deeply into the pore and lines the distal part of the conducting canal, or distal duct (Figure 2A, C). In cross-sections, distal ducts show a polymorphic outline (Figure 2C). The distal ducts pass through the cuticle of the aesthetasc pad (Figure 2B, arrowheads). Distal protrusions of the canal cells join the ducts in the middle portion of the cuticle (Figure 2D). At basal section level of the cuticle and further below, each duct is fully surrounded by at least one canal cell (Figure 2E–F). The ducts pass diagonally through the hemolymphatic space of the flagellum (Figure 2G–I).

#### Secretory cells, intermediary cells and proximal part of conducting canal

**Figure 3:**
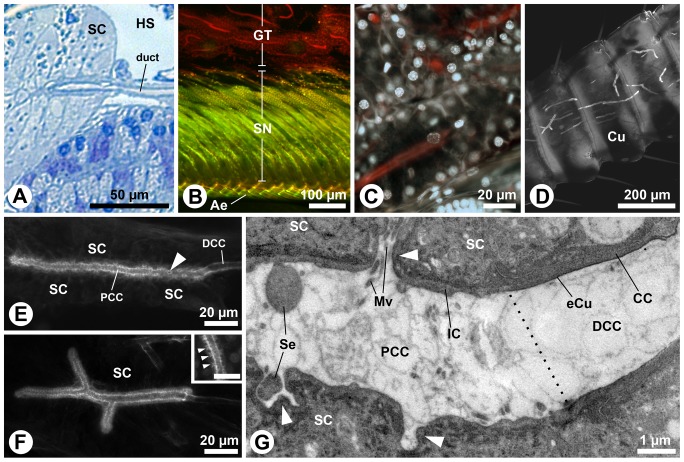
Aesthetasc-associated epidermal glands in *Coenobita clypeatus*: secretory cells, intermediary cells and the proximal duct. **A**: LM, **B-F**: cLSM, **G**: TEM. **A**: Oblique section of a duct entering the acinus. **B**: Optical sagittal section of the large flagellum with numerous proximal ducts of the aesthetasc-associated epidermal glands (red lines; phalloidin Alexa 546) located above the layer of sensory neurons cell bodies (green; sytox green). **C**: Phalloidin-labeled proximal ducts in higher detail (red; phalloidin Alexa 546), surrounded by the nuclei (white; sytox green) of the secretory cells. **D**: Antennomeres (annuli) of, proximal to the aesthetasc pad. Several proximal ducts (white lines; phalloidin Alexa 546) are visible through the cuticle. **E**: Unbranched proximal duct. **F**: Branched proximal duct; inset: higher magnification of the proximal duct showing outlets of the secretory cells (arrowheads, scale 10 µm). **G**: Longitudinal section of the proximal duct (left part of micrograph) not lined by a cuticle at the transition zone (indicated by dotted line) to the distal part of the conducting canal (right part of the micrograph) which is cuticle-lined. Note the orifices of the secretory cells opening into the proximal duct (arrowheads). Ae, aesthetasc; CC, canal cell; Cu, cuticle; DCC, distal part of the conducting canal; eCu, epicuticle; GT, glandular tissue; HS, hemolymphatic space; IC, intermediary cell; Mv, microvilli; PCC, proximal part of the conducting canal; SC, secretory cell; Se, secretion; SN, sensory neurons.

**Figure 4:**
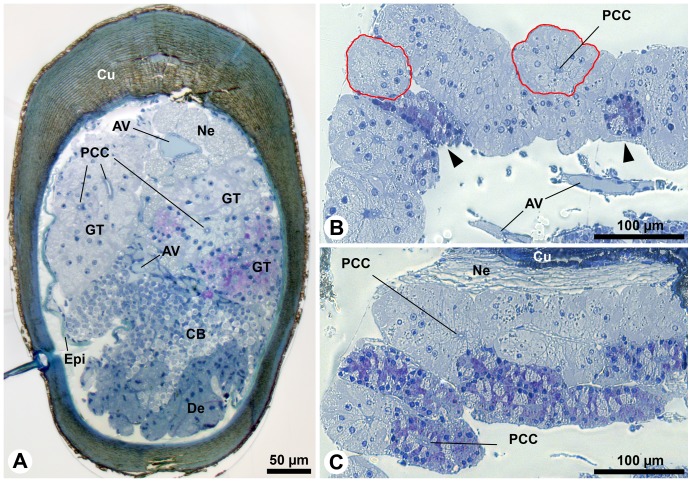
Aesthetasc-associated epidermal glands in *Coenobita clypeatus* stained with methylene blue and azur II (after Richardson et al., 1960). **A**–**C**: LM. **A**: Transverse section of large flagellum proximal to the aesthetasc pad. The epidermis has locally detached from the cuticle (in left half of the flagellum) due to a fixation artefact. The glandular tissue is located in the dorsal half of the flagellum. The ventral half is occupied by sensory neurons and associated sheath cells. **B**: Parasagittal section of glandular tissue. Note areas showing rosettes of darker stained secretory cells (arrowheads) and the nearly circular cross-sections of acini (red lines). **C**: Parasagittal section of acini. The population of darker stained secretory cells is more prominent. AV, arterial vessel; CB, sensory cell bodies; Cu, cuticle; De, dendrites of the olfactory sensory neurons; Epi, epidermis; GT, glandular tissue; Ne, antennal nerve; PCC, proximal part of the conducting canal.

The proximal part of the conducting canal (here also abbreviated as proximal duct) is formed by an intermediary cell and surrounded by numerous secretory cells. The secretory cells are clustered in a rosette-type formation, forming an elongated tubular acinus. Hence, the aesthetasc-associated epidermal glands in *Coenobita* species are of the compound tubulo-acinar type (Figure 3A–C). The acini are located above the layer of the olfactory sensory neuron cell somata; the dendrites of the latter innervate the aesthetascs (Figures 3B; 4A). The proximal ducts terminate as blind tubes inside the acini, and in different acini the proximal ducts are either unbranched (Figure 3E) or branched (Figure 3F). In high-power magnifications, outlets of the secretory cells are observed (insert Figure 3F). Notably, phalloidin staining was almost completely destroyed when incubated together with nuclear stain sytox green. The secretory cells release their products into the proximal duct (Figure 3G, arrowheads), which lacks an epicuticular intima, while the distal part of the conducting canal, lined with a thin epicuticular intima, does not have any connections to the secretory cells (Figure 3G).

**Figure 5:**
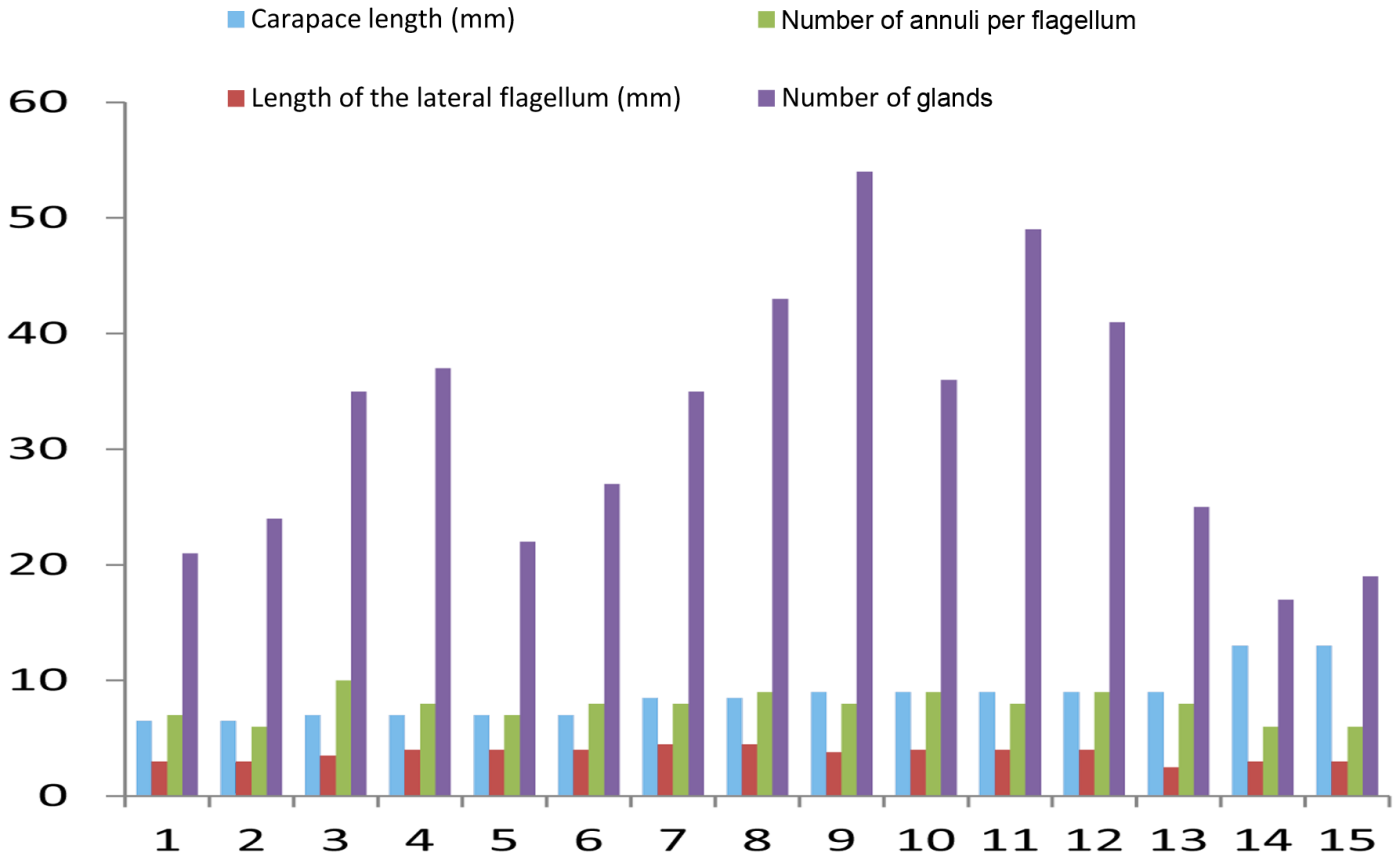
Comparative morphology and morphometry of the antennular-flagellar components in *Coenobita clypeatus*. Graph compiles length of the carapace (in mm, blue column), length of the lateral flagellum (in mm, red column), number of the annuli per flagellum (green column), and number of the proximal glandular ducts labeled with phalloidin (magenta column), compared among 15 antennules.

Methylene blue and azure II histology (according to Richardson et al. [23]) reveals two distinct types of secretory cells (Figure 4A–C). Approximately two thirds of the acini consist of secretory cells with light cytoplasm and nuclei (further referred to as acini of type 1) and one third of the acini are stained distinctly darker (type 2). We observed the acini to be monotypic, i.e. comprising the secretory cells of one type only. In transverse sections close to the aesthetasc pad, the glandular tissue occupies nearly 50% of the lumen of the large flagellum (Figure 4A). The glands can be recognized by their proximal ducts and the tubulo-acinar arrangements of the cells (Figure 4A–C). Acini have transversal diameters between 50–100 µm (Figure 4B), while they can be several hundreds of micrometers long (Figure 3B, D). Counting the total number of glandular acini in 15 antennules of *C. clypeatus* reveals enormous individual differences without any obvious relation to body size, number of annuli per flagellum or differences between females and males (Figure 5).

**Figure 6:**
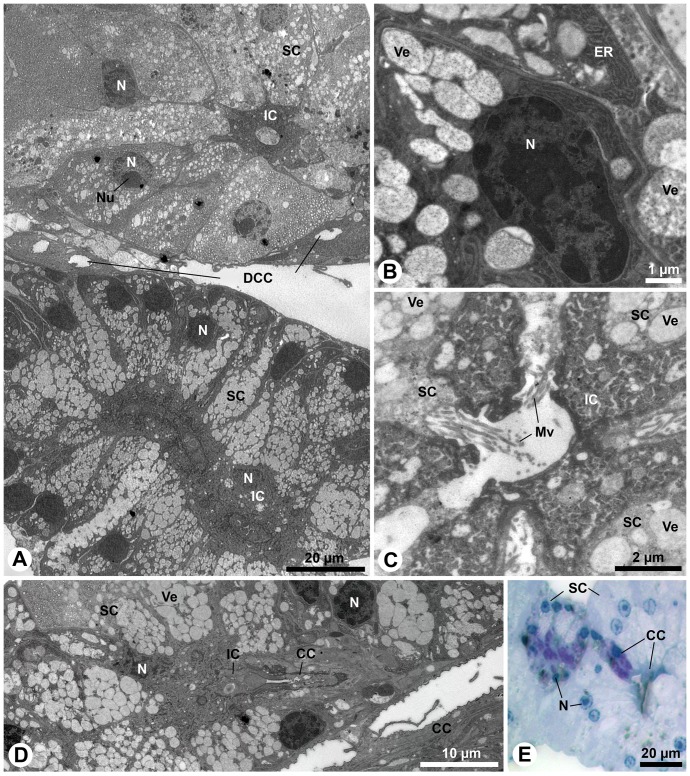
Acini in *Coenobita clypeatus*: Ultrastructure (A–D) and histological anatomy (E). **A**–**D**: TEM, **E**: LM. **A**: Oblique section showing acini of moderate (above) or strong (below) osmiophily. In both types, the secretory cells are circularly arranged around the central intermediary cell. **B**: Detail of a more osmiophilic secretory cell. Note densely aligned cisternae of the rough endoplasmatic reticulum. **C**: Transverse section of a proximal duct showing cytoplasmic details of the intermediary cell as well as apices of five surrounding, weaker electron-dense secretory cells in longitudinal section piercing the intermediary cell. Three of the secretory cells open into the proximal duct; slightly invaginated apices of the secretory cells form numerous microvilli, the latter project into the proximal duct. **D**: Oblique section showing cuticle-lined distal part of the conducting canal of a strongly osmiophilic acinus (narrow because cut tangentially), approaching another cuticle-lined distal duct. **E**: Detail showing a duct of a darker stained acinus merging into a duct of a lighter staining acinus. CC, canal cells; DCC, distal part of the conducting canal; ER, endoplasmatic reticulum; IC, intermediary cell; Mv, microvilli; N, nucleus; Nu, nucleolus; SC, secretory cell; Ve, vesicle.

Although the morphological organization of both types of acini is generally similar, several ultrastructural differences exist and at least two of them are elucidated in the different staining results of the secretory cells visible in histological sections. Secretory cells with light cytoplasm have large, spherical nuclei with light caryoplasm and prominent nucleoli (Figure 6A). The nuclei are situated approximately in the centre of the cells (Figures 4B–C, 6A). The cytoplasm is densely packed with relatively small vesicles filled with an electron-lucent substance, most probably secretion (Figure 6A). The nuclei of the secretory cells with stronger osmiophilic cytoplasm are somewhat smaller (Figure 6A–B). They are predominantly located in the basal portion of the cells (Figures 4B; 6A). Their nucleoli are inconspicuous and hardly visible. The cytoplasm is packed with relatively large vesicles filled with a slightly less electron-lucent secretion and with dense endoplasmic reticulum (ER), especially close to the nuclei (Figure 6A–B). All secretory cells have a conical, columnar shape with heights of 30–50 µm, basal diameters of 10–20 µm and apical diameters of 1–2 µm. Because of their alternating arrangement around the central intermediary cell, 10–15 cell bodies can be counted per transverse section (Figure 6A). The intermediary cells have average diameters of 10–20 µm (Figure 6A). The narrow apices of the secretory cells pass through the intermediary cells (Figure 5C). Since the proximal ducts are only about 5 µm wide, a maximum of 6 secretory cells can release their products into the duct lumen at the same transverse section level of the lumen (see Figure 6C). The secretory cells possess typical apical microvilli borders but no other peculiar apical structures like reservoirs, fibrous sieves or valves (Figure 6C). The microvilli are several micrometers long and can reach the centre of the lumen (Figure 6C).

In both types of acini, the secretion is first collected in the proximal duct and then transferred to the cuticle-lined distal part of the conducting canal (Figure 3G moderately osmiophilic (type 1) secretory cells; Figure 6D strongly osmiophilic (type 2) secretory cells). Histological evidence show that distal ducts of type 2 acini approach and merge into the distal ducts originating from type 1 acini (Figure 6D, E).

### CUB-serine protease immunoreactivity

**Figure 7:**
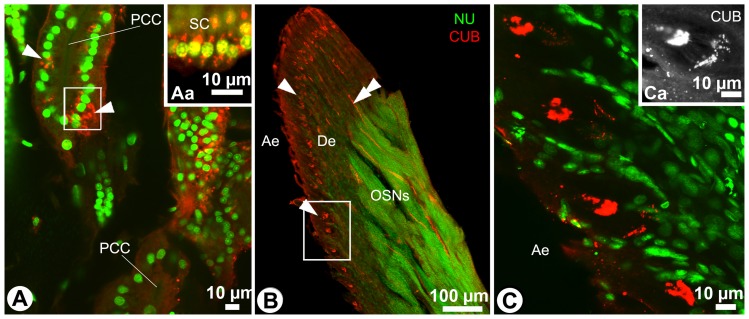
CUB-immunoreactivity in the antennules of *Coenobita clypeatus*. Flagellar tissues labeled with antibodies against the CUB domain of serine protease identified in the spiny lobster *Panulirus argus* (CUB, red) and stained with the nuclear marker sytox green (NU, green). cLSM. **A**: CUB-immunoreactivity in the secretory cells (arrowheads), **Aa**: some secretory cells from A (white frame) at higher magnification. **B**: An overview of the whole flagellum. The arrowheads mark the enlargements formed by sheath cells surrounding the dendrites of olfactory sensory neurons, the double arrowhead points towards labeling in the distal part of the conducting canal. **C**: CUB-immunoreactivity in the enlargements formed by sheath cells surrounding the dendrites of olfactory sensory neurons, **Ca**: details of CUB-immunoreactivity visualized in single (red) channel. Ae, aesthetasc field; De, dendrites of olfactory sensory neurons (OSNs); PCC, proximal parts of the conducting canal.

Treatment of the antennules with the antibody against the CUB domain of *Panulirus argus* serine protease (Csp) resulted in labeling of the glandular tissue in *Coenobita clypeatus* (Figure 7). CUB-immunoreactivity was observed within the secretory cells in the form of asymmetrical aggregations measuring 0.5–2 µm in diameter (Figure 7A, arrowheads). Not all secretory cells within a single acinus were CUB-immunoreactive, and it was not possible to differentiate between the two types of secretory cells using counterstaining with nuclear marker sytox green. Most of the CUB-immunoreactivity was concentrated close to the nucleus (Figure 7Aa). Notably, the proximal ducts lack CUB-immunoreactivity (Figure 7A), while at least parts of the distal ducts were labeled distinctly (Figure 7B, double arrowhead). In addition to the glandular tissue, CUB-immunoreactivity was detected at the level of the cilia formation, where the sheath cells surrounding the dendrites of the olfactory sensory neurons form an enlarged receptor lymph space around the cilia (Figure 7C, Ca; morphology described in e.g., [8], [29]). No immunoreactivity was detected in the auxiliary cells (sheath cells surrounding the inner dendritic segments of the olfactory sensory neurons) of *Coenobita*, or any other antennular tissue. Negative controls, treated with 0.5% NGS PBS-Tx only, did not show any specific fluorescent signal.

**Figure 8:**
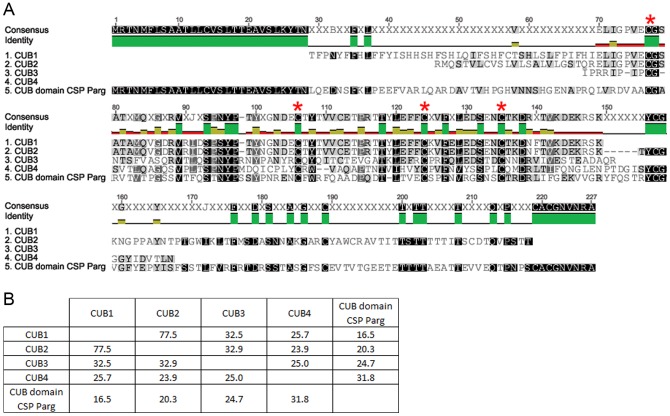
CUB domain in *Coenobita clypeatus* ESTs. **A**: MUSCLE multiple sequence alignment of four putative CUB domain containing ESTs after translation into amino acids. Reference sequence: CUB domain of the Csp of *Panulirus argus* (Schmidt et al., 2006). Conserved cysteine residues are indicated by red asterisks. **B**: Similarity matrix of sequences, values are given in percent.

To test whether or not CUB-serine protease is expressed in the antennules of *C. clypeatus* BLAST searches against the translated nucleotides of the *C. clypeatus* antennal transcriptome were performed [26]. We identified four contigs similar to the CUB-domain and several contigs matching the trypsin-domain of the reference sequence of *Panulirus argus*. However, the contigs were not spanning the entire sequence of the CUB domain serine protease. As several trypsin-like serine proteases are to be expected in the tissue we focused only on those ones matching the CUB domain. Figure 8A displays a multiple sequence alignment of the 4 identified putative CUB domain containing contigs. The amino acid similarity to the reference Csp of *P. argus* varied between 16.5% and 31.8% and was highest in the putative *C. clypeatus* CUB4. The similarity of putative *C. clypeatus* CUBs varied between 23.9% and 77.5% (see similarity matrix, Figure 8).

### Protein identification

To perform proteomic analysis the soluble proteins of glandular complexes-containing tissue and antennular tissue containing olfactory sensory neurons were subjected for LC-MS/MS analysis. In order to identify protein candidates characterizing the gland secretions we compared the proteome of glandular complexes-containing tissue with those of the antennular tissue containing olfactory sensory neurons. As the genome of *C. clypeatus* is not available we used an in-house constructed transcriptome of *C. clypeatus* antennules and processed the acquired tandem mass spectra using combined proteomic strategy. The first step was to search them against available protein databases to identify proteins from the *C. clypeatus* protein subdatabase or to match peptides from highly conserved protein domains of closely related species (stringent database searching) followed by homology-based protein identification that relies on *de novo* sequencing of the acquired MS/MS spectra and searching them against available databases using mass spectrometry-driven BLAST [30] to identify proteins by homology (error-tolerant searching). To increase sequence coverage of the analyzed proteins we additionally applied data-independent acquisition referred to as MSE [30] which complements conventional data-dependent acquisition providing independent means of data validation.

**Figure 9:**
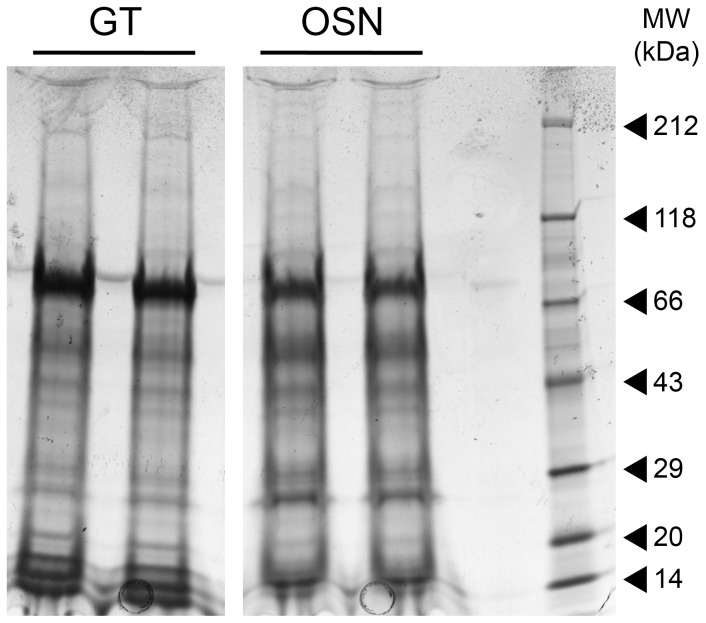
1-D SDS-PAGE profiles of the proteomes of two samples tested. Protein bands were visualized with Coomassie Blue (R250). GT, glandular tissue; OSN, olfactory sensory neurons containing tissue.

The complete lists of the proteins identified in glandular complexes-containing tissue (GT) and antennular tissue containing olfactory sensory neurons (OSN) are depicted in (Tables S1, S2). Major bands for both samples ranked between 10 and 66 kDa, with the first strong band appearing at ca. 66 kDa and corresponding to ubiquitous proteins such as hemocyanin and heat shock proteins (Figure 9). All these hits along with other high abundant proteins such as beta-actin, alpha and beta tubulin, histone complex subunits as well as related contigs from the *C. clypeatus* antennal transcriptome [26] were mostly matched by all applied protein identification workflows. The major differences in the protein pattern were observed in the mass range from 14 to 20 kDa. Table 1 summarizes the annotated proteins only present in the glandular tissue (full list of proteins only present in glandular tissue in (Table S3). Most of these proteins indicate involvement in the secretory pathway, i.e. taking part in intracellular transport between the endoplasmatic reticulum (ER) and the Golgi apparatus, within the Golgi cisternae and the specific addressing and processing of vesicles. A second group is putatively involved in immune responses, including serine and aspartate proteases (coagulation factor XI, cathepsin D, hydrolase), protease inhibitors (alpha 2 macroglobulin), lectins, enzymes involved in responses to reactive oxygen species (such as glutathione-S-transferases) and an enzyme activated by interferon gamma (gamma-interferon inducible lysosomal thiol reductase). One identified protein is homologous to an uncharacterized protein produced in the salivary glands of *Ixodes scapularis* (Say, 1821). No CUB-serine proteases or CUB domain alone were identified in the proteome, neither in the glandular tissue, nor in the control sample.

**Table 1:** Proteins exclusively present in the tissue containing aesthetasc-associated epidermal glands in *C. clypeatus*.

Protein	Mascot				MS BLAST				MSE				GO association
	Accession	Species	Score	Peptide hits	Accession	Species	Score	Peptide hits	Accession	Species	Score	Peptide hits	
alpha 2 macroglobulin (ConsensusfromContig31628_full_rev_frame_1)						*Coenobita*	112	2		*Coenobita*	2516	3	GO∶0019731: antibacterial humoral response
alpha 2-macroglobulin					ABD6146	*Scylla serrata*	278	6					
alpha spectrin	EFX88672	*Daphnia pulex*	296	6	XP_002083280	*Drosophila simulans*	617	13					
aspartate protease cathepsin D									AEO94539	*Triatoma infestans*	520	2	GO∶0008233: peptidase activity
coagulation factor XI					XP_001845410	*Culex quinquefasciatus*	72	1					GO∶0006508∶proteolysis
coatomer protein complex					NP_001166195	*Bombyx mori*	71	1					GO∶0016192∶vesicle-mediated transport
coatomer protein complex (ConsensusfromContig810 full rev frame 0)										*Coenobita*	639	2	
coatomer protein delta, isoform B									NP_001162642	*Drosophila melanogaster*	723	5	
coatomer subunit delta-like									XP_003700942	*Megachile rotundata*	738	6	
C-type lectin (ConsensusfromContig110279_full_fwd_frame_0)		*Coenobita*	82	1						*Coenobita*	1730	1	GO∶0002682∶regulation of immune system process
emp24 cargo transport protein (ConsensusfromContig108988_full_fwd_frame_0)										*Coenobita*	307	2	GO∶0051050∶positive regulation of transport
gamma-interferon inducible lysosomal thiol reductase					XP_002400600	*Ixodes scapularis*	65	1					GO∶0007166∶cell surface receptor signaling pathway
GH15863 gene product from transcript GH15863-RA, Peptidase_C26					XP_001983370	*Drosophila grimshawi*	105	2					GO∶0016787: hydrolase activity
GL17639 (ConsensusfromContig35747 full rev frame 1)										*Coenobita*	321	3	GO∶0000042∶protein targeting to Golgi
glutathione-S-transferase (ConsensusfromContig523_full_rev_frame_0)						*Coenobita*	77	1					GO∶0006979∶response to oxidative stress
glutathione-S-transferase (ConsensusfromContig80526_full_rev_frame_0)										*Coenobita*	426	3	
glutathione-S-transferase (ConsensusfromContig83104_full_rev_frame_0)										*Coenobita*	765	1	
GST_N_Metaxin									XP_002430330	*Pediculus humanus corporis*	489	4	GO∶0070122: isopeptidase activity
S-formylglutathione hydrolase (ConsensusfromContig31027 full rev frame 1)										*Coenobita*	392	1	GO∶0018738∶S-formylglutathione hydrolase activity
signal peptidase complex subunit 3-like					XP_003739905	*Metaseiulus occidentalis*	65	1					GO∶0009003: signal peptidase activity
sorting nexin-12									XP_002432737.1	*Pediculus humanus corporis*	841	3	GO∶0015031∶protein transport
trafficking protein particle complex subunit 10									XP_003742750	*Metaseiulus occidentalis*	671	2	GO∶0016192: vesicle-mediated transport
transient receptor potential-gamma protein									EFN78599	*Harpegnathos saltator*	439	1	GO∶0005261: cation channel activity
uncharacterized protein									XP_003748091	*Metaseiulus occidentalis*	531	2	homology “secreted salivary gland peptide” Isca XP_002414539

## Discussion

### Characterization of aesthetasc-associated epidermal glands

Epidermal (tegumental) glands of crustaceans exhibit a wide range of structural complexities and are widespread in the crustacean integument. However, literature does only cover a fraction of the morphological diversity of epidermal glands in this group. Some crustacean taxa received attention with respect to their epidermal glands (e.g., Notostraca: [31]; Decapoda: [13], [21]; Peracarida: [32], [33]), while others remained rather disregarded, even though considered key groups in the debate on arthropod and crustacean phylogeny (e.g., Remipedia). Moreover, previous accounts addressed epidermal glands of certain body regions, in particular the head and appendages. For instance, previous studies gave insights into the anatomy of intra- and subepidermal glands of the head and mouth parts in land isopods [34], as well as pleopods [35], eyestalk [36], foregut, hindgut and the gill chamber [37] and antennae [13], [38] in aquatic decapods. The epidermal glands are ubiquitous in some species, whereas in others they are often sparse and restricted to certain locations [39], [20]. Epidermal glands in isopods have been shown to be especially abundant and structurally variable in terrestrial species compared to aquatic ones [37], and it has been suggested that ubiquity of epidermal glands might be connected to terrestrial adaptation in these animals [34], [40], [41].

Several reports mention pore structures potentially associated with different types of antennal sensilla of decapod crustaceans (summarized by [13]), but few studies linked external morphology of pore structures to electron microscopic data on cellular apparatus associated with these kinds of pores. Examples for these rare comprehensive approaches are the investigations on unicellular epidermal glands associated with guard setae in *Homarus gammarus* (Linnaeus, 1758) (see [42]),“rosette-type tegumental glands” associated with olfactory sensilla (aesthetascs) in *Panulirus argus* [13] and tegumental glands in the olfactory organ of *Homarus americanus* [38]. Even less information is available on the composition of the secretory product and the possible function of these glands in antennules.

Aesthetascs of the spiny lobster *P. argus* are numerous, long and slender setae [29], accompanied by other, non-olfactory sensilla: guard, companion and asymmetric setae [43]. Aesthetascs in *Coenobita* appear to have undergone specific adaptations to the terrestrial habitat: they are short and blunt [8]; [44], with slender and minute non-olfactory setae occurring mainly on the margins of the aesthetasc pad. The pores surrounded by characteristic collar-like folds, associated with the aesthetascs of *Coenobita*, however, resemble the “peg pores” of the aesthetasc tegumental glands (ATGs) identified in the spiny lobster *P. argus* [13]. In contrast, no structural analog to the more sparsely arranged minute “depression pores” [13] could be identified in the aesthetasc pad of *Coenobita*. Neither could unicellular glands like those known for *Homarus* (e.g. [42]) be observed in the area of the *Coenobita* aesthetasc pad. However, solitary minute pores which may resemble the “depression pores” [13] could be observed outside the aesthetasc pad in *C. clypeatus*. ATGs in *P. argus* lay directly beneath the cuticle of the large flagellum [13], while the aesthetasc-associated epidermal glands of *Coenobita* spp. are sunk much deeper into the inner regions of the flagellum. This substantial shift of the glands results in a considerable elongation (several hundreds of micrometers) of the distal glandular ducts. Organs being this much distanced from the cuticular surface and the subjacent epidermis, may have been an important prerequisite to live on land, as deeply sunk organs are prevented from desiccation and/or overheating (compare [45]). The aesthetasc-associated epidermal glands in *Coenobita* are bigger in diameter and longer (more flask-like rather than rosette-like), with more secretory cells per acinus. Bigger glands suggest that higher volume of secretion need to be produced in a short period of time, i.e. to impregnate the delicate cuticular shafts of the aesthetascs, and to react quickly to changes in air humidity.

The results of this study reveal that the general architecture of epidermal glands described for many different crustacean taxa (see review [20]) applies to hermit crabs (Paguroidea) as well. Epidermal glands of *Coenobita* spp. share typical basic set-up (class-III-glands acc. to [17], subclass of recto-canal epidermal glands acc. to [18]), comprising three cell types: secretory cells, which are organized in a rosette around the non-cuticularized proximal duct, intermediary cells (usually one intermediary cell per acinus), and canal cells, the latter forming the distal cuticularized part of the conducting canal. However, epidermal glands of *Coenobita* spp. also differ from those of other crustaceans by their higher complexity. The secretory cells comprise two types, which stain differently according to the protocol of Richardson et al. [23]. This effect, known as metachromasia, can be explained by different binding patterns of the dyes to substances in the tissue characterized by different chemical properties. In case of epidermal glands the reasons for metachromasia are the chemistry of the secretory material, as well as ultrastructural differences, such as variations in the density of rough endoplasmic reticulum. The fusion of the distal ducts from the acini with these two different secretory cell types indicates that their secretory products get mixed before being released onto the surface of the aesthetasc pad. Notably, the proximal ducts in *Coenobita* spp. being narrow and often branched are quite different from the proximal ducts of ATGs of their aquatic relative *P. argus* [13]. In the latter species, the proximal ducts resemble the local enlargements of the conducting canal, i.e. reservoirs, and never branch.

Investigation of proteomes in non-model organisms using similarity-based searching (MS BLAST) has been successfully used in many studies [29]. However, it still remains a challenging task characterizing species with significant phylogenetic diversity and the success rate of identification dramatically decreases with evolutionary distance to its closest relative represented in the databases [30]. While data from insecta clade within Pancrustacea [46], are well represented in the available databases, molecular data from crustaceans is sparse and lead to a high number of proteins found in the antennal transcriptome and antennal proteome but lacking matches to described homologues in other species. The uncharacterized transcripts listed in Table S3 might play important roles in the function of the epidermal glands but need further characterization. The peptides identified in the epidermal glands of *Coenobita* indicate a classic merocrine pathway from precursors produced in cisternae of rough ER, transportation to and processing in Golgi bodies, vesicular budding, collection in fusion granules, and final extrusion of secretion at the apex of the secretory cell (see Table1). The abundance of rough ER cisternae in epidermal glands suggests that glycoproteins might be a major secretory product [20]. The proximal ducts of the epidermal glands can be visualized with phalloidin, and thus the intermediary cell forming the lumen and/or secretory cells contain high concentrations of F-actin filaments. A similar phalloidin-positive staining was described in pleopod tegumental glands of the American lobster *Homarus americanus* (H. Milne Edwards, 1873) [21] and ATGs of *P. argus* [13]. The absence of phalloidin staining after incubation of the tissues with nuclear stain sytox green is probably due to an interaction with dimethyl sulfoxide. There are cases known when the intensity of phalloidin labeling is also affected by heat [13]. Rich networks of actin filaments can provide mechanical support for the proximal duct, help to maintain its shape and prevent the lumen from collapsing. It is also possible that filamentous actin together with myosin can function in order to push secretory products into the distal part of the conducting canal of the gland; we however did not see any evidence for myosin filaments or microtubules inside the intermediary cells forming the proximal duct, so it might be sufficient if the secretory products simply flow through the system. The distal parts of the conducting canal cannot be visualized with phalloidin. This makes us suggest that some additional mechanisms might be involved in guiding the secretion through the distal duct system and facilitating the final release of the glandular products onto the surface. It is known that secretion is released during antennular grooming when the first antennae are clasped and smeared over repeatedly by maxillipedes in the spiny lobster [47]. In some arthropods, muscles have been found in association with the epidermal glands, however not in the antennae [48]. Anyway, pulsating hemolymphs, propelled by the circulatory system, might also influence transport and release of secretions due to compressing the acini and the ducts. Since the secretory cells of the aesthetasc-associated epidermal glands of *Coenobita* neither possess any storage compartments or reservoirs larger than regular secretory granules, nor any kind of valve-like structure regulating the secretion flow, it is likely that as long as the glands deliver their product into the ducts, a constant flow of highly fluid secretion will reach the surface.

The mechanism that regulates and activates secretion in epidermal glands is poorly understood. It is assumed that in general crustacean epidermal glands are not innervated [20], although some exceptions have been reported, like the rosette-type glands in the gills of the grass shrimp *Palaemonetes pugio* (Holthuis, 1949), where synapses have been observed on the secretory cells [49]. Treatment of *Coenobita* antennules with synapsin antibodies did not reveal any positive immunoreactivity (Tuchina et al., unpublished data), suggesting that antennal epidermal glands in *Coenobita* are not innervated and the secretion is triggered and regulated by some other mechanism. Many epidermal glands have been shown to synchronize their secretion activity with the molting cycle [50], [51], which means they are probably hormonally regulated. However, no such hormones have been identified to be produced in epidermal glands of coenobitids so far.

### Functions of aesthetasc-associated epidermal glands and serine proteases

Although epidermal glands can be structurally similar at different body regions such as mouth parts and antennae, their presumed function, i.e. histochemistry of the secretory product, is likely different. Epidermal glands in the legs of the terrestrial isopod *Armadillium vulgare* (Latreille, 1804) produce polyphenol oxidase, an enzyme that has been implicated in the tanning of the cuticle after molting [52]. Epidermal glands from the gut of the lobster *Homarus gammarus* (Linnaeus, 1758) appear to contain phosphatases, ATPases and mucopolysaccharides [53], glands found beneath the cuticle of the head and mouth parts are often referred to as salivary glands or thought to be involved in digestion [20], while in glands of the amphipod *Gammarus pulex* (Linnaeus, 1758) catecholamines, like for example dopamine, seem to be the major secretory product [54].

The antennal epidermal glands of the spiny lobster *Panulirus argus* have been shown to contain specific type of proteases, CUB-serine proteases (Csp) [16], [55]. In the following studies by Schmidt et al. [13] it was confirmed that Csps are found exclusively in the secretory cells of aesthetasc tegumental glands, ATGs, and are likely secreted onto the surface of the aesthetascs. A serine protease with low sequence similarity to Csp but with similar distribution pattern as in *P. argus* was also found in the antennae of *H. americanus* [56] with one olfactory enriched transcript (OET-03), identified as chymotrypsin-like serine protease and exclusively expressed in the secretory cells of aesthetasc-associated tegumental glands [38]. By homology searches we identified four putative CUB domains in the antennal transcriptome of *C. clypeatus*. The ESTs were short and did not allow a final characterization; however we find the four cysteine residues at their fixed positions as reported by Levine et al. [16] for *P. argus*. The reason why we did not find Csp or CUB domain alone in the antennal gland proteome might be because the proteins were not separated perfectly, and it is especially likely to be the case for membrane-associated proteins.

Serine proteases are known to have manifold functions. Thus, their role in the crustacean olfactory system is uncertain and a matter of ongoing discussion. It has been shown that serine proteases and their inhibitors are involved in the development of the nervous system, including cellular migration, differentiation, process extension, as well as repairing and apoptosis of neurons and glial cells in vertebrates [57], [58]. The pattern of activity of Csps in the antennae of the spiny lobster varies along the developmental axis of the olfactory organ, suggesting a possible role of Csps in development, maturation and/or degradation of olfactory sensory neurons [55], although the Csp-immunoreactivity was detected exclusively in glandular tissue [13]. In *Coenobita*, we identified CUB-immunoreactivity in the enlargements formed by the sheath cells surrounding the dendrites of the olfactory sensory neurons (not to be mixed with the report by Levine et al. [16] about Csp-immunoreactivity in auxillary cells in *P. argus*). The enlargements surrounding olfactory cilia in *Coenobita* spp. have been previously reported by Ghiradella et al., [59] and assumed to help in maintaining the proper osmotic conditions around cilia and also probably serve as mechanical shock absorbers. As reported by Schmidt et al., [13] for *P. argus*, the distribution of immunoreactive material was seemingly identical for two antisera used in their study: no. 99-6 raised against the CUB domain and 102-6 raised against the protease domain. In this study, only 5th bleed serum (99-5) against the CUB domain of the Csp was tested. It can be extrapolated that distribution of the Csp within the glandular cells most likely also corresponds to the detected CUB-immunoreactivity. The function of Csp in association with the dendrites of olfactory sensory neurons is unclear, but the possibility that serine proteases might be involved in development, apoptosis or maintenance of OSN dendritic function in *Coenobita* cannot be excluded and needs further investigation.

Other functions of serine proteases include perireception, regulation of other proteins and hormones, blood clotting, and immune response [60], [61]. The serine proteases from ATGs of the spiny lobster *P. argus* have been suggested to be involved in enzymatic degradation of odorant molecules and/or production of active odorants from inactive precursors [60], [62]. Peptide-mediated behaviors are well documented in aquatic animals [60]. Short peptides, free amino acids and their binary mixtures are known to trigger specific behaviors in crustaceans, such as attraction of anomuran crabs to the new shells [63], [64], and simultaneous release of larvae and induction of larval settlement behavior in brachyuran crabs [65]. Non-volatile peptide cues from a dead snail have been shown to attract marine hermit crabs within minutes [66]. Females of the estuarine mud crab *Rhithropanopeus harrisii* (Gould, 1841) perform rhythmic and highly synchronized patterns of larval release (‘abdominal pumping’) in a response to carboxyl terminal arginine peptides (‘pumping factor’), the latter are generated by the action of trypsine-like serine proteases on the membranes of the hatching eggs [67]. A pumping response can also be evoked, to a different degree, by treatment with exogenous trypsin, trypsin inhibitors [67] or a mixture of arginine and glycine, the two amino acids that are most prevalent after hydrolysis of the pumping factor [68]. As for hermit crabs, several amino acids, including arginine, glycine and leucine, were screened in *Pagurus bernhardus* (Linnaeus, 1758) and *C. clypeatus*, but did not elicit any response in electro-antennographic recordings [6], and thus were not tested behaviorally.

Aesthetasc-associated epidermal glands in *Coenobita* might play a role in chemosensing, the main function of crustacean antennules, however, no odorant binding proteins were found in proteomic analysis, or in the antennal transcriptome of *C. clypeatus* [26]. We also observed that epidermal glands in *Coenobita* are present in similar numbers in both sexes so we consider it unlikely that the secretions have a role in sexual communication. Schmidt and coauthors [13] proposed that epidermal glands secretion in *P. argus* have anti-fouling and/or friction-reducing properties, and would thus be important for the proper functioning of the aesthetascs. This hypothesis is corroborated by our data for *Coenobita* spp. Crustaceans, like other invertebrates do not possess acquired immunity and have to rely solely on the innate immune system as a defense against invading microorganisms. Aesthetascs with their thin cuticle are likely to be easy targets for microbial invaders, and in fact the antennular tissue in *C. clypeatus* was identified as a place of production of antimicrobial substances indicating the presence of a highly diverse microbial community [26]. Moreover, Krång et al. [6] demonstrated that high humidity is critical for odor perception in *C. clypeatus* and putatively also enhances microbial growth. The innate immune responses in crustaceans include melanization by activation of prophenoloxidases, a clotting process, phagocytosis, encapsulation and cell agglutination [61]. The components of the innate immune system, such as coagulation factor XI, cathepsin D and alpha 2 macroglobulin were identified in the proteome of the gland secretions, as well as diverse enzymes, involved in the responses to oxidative stress (catalases, glutathione reductases, glutathione-S-transferases, superoxide dismutases and peroxiredoxins). Opaque granules of melanin pigments were frequently observed in the antennular tissue of *C. clypeatus* (Tuchina et al., unpublished data) – an indication of a melanization process, a common reaction to pathogens among arthropods. The innate immune system is usually represented by its humoral (antimicrobial factors circulating in hemolymph) and cellular (hemocytes) components, so we find the need of a specialized structure, such as antennular epidermal glands producing a particular type of serine proteases in several crustacean species, is very remarkable.

We propose that the aesthetasc-associated epidermal glands in *Coenobita* spp. produce a mucous-like substance covering the aesthetascs and providing both moist interface essential for odor perception as well as mechanisms for antimicrobial defense, such as CUB-serine proteases, and probably other components of innate immunity. Together with the morphological characteristics of the glands, the proposed functions of the mucous are important adaptations for the functionality of the aesthetasc sensory system within terrestrial environments.

## Conclusion

Olfactory sensilla in *Coenobita* spp. are covered by mucous-like substance, which is produced by aesthetasc-associated epidermal glands of high morphological complexity. The mucous takes part in antimicrobial defense and at the same time provides moisture which is known to be critical for odor perception in terrestrial hermit crabs. The morphological modifications of the aesthetasc-associated epidermal glands as well as the functional characteristics of their secretions are important adaptations to a terrestrial lifestyle.

## Supporting Information

Table S1 **The complete list of proteins identified in the glandular complexes-containing tissue, GT.**
(XLSX)

Table S2 **The complete list of proteins identified in the antennular tissue containing olfactory sensory neurons, OSN (control sample).**
(XLSX)

Table S3 **The complete list of proteins present exclusively in the glandular tissue.**
(XLSX)
